# 2-Bromo-1-(4-methyl­phen­yl)-3-phenyl­prop-2-en-1-one

**DOI:** 10.1107/S1600536808022289

**Published:** 2008-07-19

**Authors:** Hoong-Kun Fun, Samuel Robinson Jebas, P. S. Patil, M. S. Karthikeyan, S. M. Dharmaprakash

**Affiliations:** a X-ray Crystallography Unit, School of Physics, Universiti Sains Malaysia, 11800 USM, Penang, Malaysia; b Department of Studies in Physics, Mangalore University, Mangalagangotri, Mangalore 574 199, India; c Syngene International Pvt Limited, Plot Nos. 2 and 3 C, Unit-II, Bommansandra, Industrial Area, Banglore 560 099, India

## Abstract

In the crystal structure of the title compound, C_16_H_13_BrO, the two benzene rings are twisted from each other with a dihedral angle of 52.55 (9)°. Both an intra­molecular C—H⋯Br hydrogen bond, which generates an *S(*6) ring motif, and a short Br⋯O contact [2.9907 (19) Å] may influence the conformation of the mol­ecule. The crystal packing is stabilized by weak inter­molecular C—H⋯O inter­actions.

## Related literature

For related literature on chalcone derivatives, see: Fun *et al.* (2008 ▶); Patil *et al.* (2006 ▶, 2007 ▶). For related literature on experimental preparation, see: Shivarama Holla *et al.* (2006 ▶). For standard bond-length data, see: Allen *et al.* (1987 ▶). For graph-set analysis of hydrogen bonding, see: Bernstein *et al.* (1995 ▶).
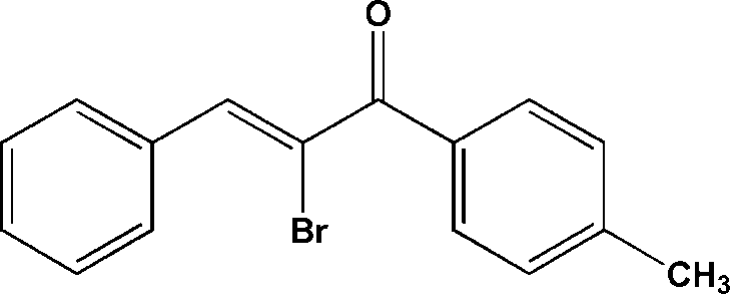


## Experimental

### 

#### Crystal data

C_16_H_13_BrO
*M* *_r_* = 301.17
Orthorhombic, 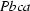

*a* = 8.7192 (2) Å
*b* = 11.5819 (2) Å
*c* = 26.4769 (6) Å
*V* = 2673.77 (10) Å^3^
*Z* = 8
Mo *K*α radiation
μ = 3.06 mm^−1^
*T* = 100.0 (1) K
0.20 × 0.20 × 0.11 mm

#### Data collection

Bruker SMART APEXII CCD area-detector diffractometer
Absorption correction: multi-scan (*SADABS*; Bruker, 2005 ▶) *T*_min_ = 0.556, *T*_max_ = 0.715
14370 measured reflections
3893 independent reflections
2462 reflections with *I* > 2σ(*I*)
*R*_int_ = 0.070

#### Refinement

*R*[*F* ^2^ > 2σ(*F* ^2^)] = 0.041
*wR*(*F* ^2^) = 0.090
*S* = 1.00
3893 reflections
164 parameters
H-atom parameters constrained
Δρ_max_ = 0.43 e Å^−3^
Δρ_min_ = −0.54 e Å^−3^

### 

Data collection: *APEX2* (Bruker, 2005 ▶); cell refinement: *APEX2*; data reduction: *SAINT* (Bruker, 2005 ▶); program(s) used to solve structure: *SHELXTL* (Sheldrick, 2008 ▶); program(s) used to refine structure: *SHELXTL*; molecular graphics: *SHELXTL*; software used to prepare material for publication: *SHELXTL* and *PLATON* (Spek, 2003 ▶).

## Supplementary Material

Crystal structure: contains datablocks global, I. DOI: 10.1107/S1600536808022289/lh2662sup1.cif

Structure factors: contains datablocks I. DOI: 10.1107/S1600536808022289/lh2662Isup2.hkl

Additional supplementary materials:  crystallographic information; 3D view; checkCIF report

## Figures and Tables

**Table 1:** Hydrogen-bond geometry (Å, °)

*D*—H⋯*A*	*D*—H	H⋯*A*	*D*⋯*A*	*D*—H⋯*A*
C1—H1*A*⋯O1^i^	0.93	2.54	3.163 (3)	124
C11—H11*A*⋯Br1	0.93	2.69	3.377 (3)	131

Symmetry code: (i) 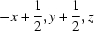.

**Fig. 1.**
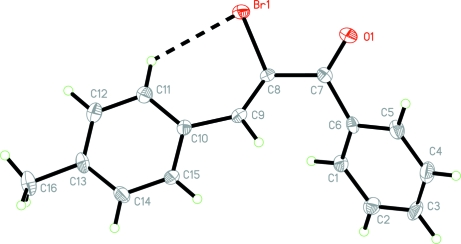
The molecular structure of the title compound, showing 50% probability displacement ellipsoids and the atom numbering scheme.

**Fig. 2.**
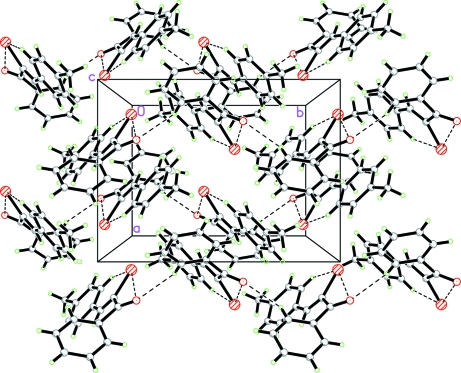
The crystal packing of the title compound, viewed along the c axis. Hydrogen bonds and Br···O short contacts are shown as dashed lines.

## supplementary crystallographic information

### Comment

As part of our crystallographic studies on chalcone derivatives (Fun *et al.*, 2008; Patil *et al.*, 2006,2007) the title compound (I) was synthesized and its crystal structure is reported here.

In the crystal structure of the title compound (I), the bond lengths have have normal values (Allen *et al.*, 1987). The two benzene rings (C1—C6 & C10—C15) are twisted from each other with the dihedral angle of 52.55 (9)°.

Both an intramolecular C—H··· Br hydrogen bond, which generates an S(6) ring motif, and a short Br···O =2.9907 (19)Å contact may influence the conformation of the molecule. The crystal packing is stabilized by weak C—H···O intermolecular interactions.

### Experimental

1-(4-methylphenyl)-3-phenylprop-2-en-1-one (1 mmol) was prepared by a literature procedure (Shivarama Holla *et al.*, 2006). To a solution of 1- (4-methylphenyl)-3-phenylprop-2-en-1-one (1 mmol) in chloroform (25 ml), bromine (1 mmol) was added slowly with stirring. After the completion of addition of bromine (1 mmol), the reaction mixture was stirred for 24 h. Excess of chloroform was distilled off and the precipitated 2,3- dibromo-1-(4- methylphenyl)-3-phenylpropan-1-one was filtered off and dried. A mixture of dibromopropanone (1 mmol) and triethylamine(1 mmol) in dry benzene (30 ml) was added and the resultant mixture was stirred for 24 h. The excess of solvent when removed under reduced pressure gave the title compound which crystallized from acetone by slow evaporation.

### Refinement

H atoms were positioned geometrically [C—H = 0.93Å and CH_3_=0.96 Å] and refined using a riding-model, with *U*_iso_(H) = 1.2*U*_eq_(C) and 1.5_eq_(C_methyl_). A rotating group model was used for the methyl groups.

### Crystal data

**Table:**

C_16_H_13_BrO	*F*_000_ = 1216
*M**_r_* = 301.17	*D*_x_ = 1.496 Mg m^−^^3^
Orthorhombic, *P**b**c**a*	Mo *K*α radiation λ = 0.71073 Å
Hall symbol: -P 2ac 2ab	Cell parameters from 1664 reflections
*a* = 8.7192 (2) Å	θ = 2.8–23.7º
*b* = 11.5819 (2) Å	µ = 3.06 mm^−^^1^
*c* = 26.4769 (6) Å	*T* = 100.0 (1) K
*V* = 2673.77 (10) Å^3^	Block, colourless
*Z* = 8	0.20 × 0.20 × 0.11 mm

### Data collection

**Table:**

Bruker SMART APEXII CCD area-detector diffractometer	3893 independent reflections
Radiation source: fine-focus sealed tube	2462 reflections with *I* > 2σ(*I*)
Monochromator: graphite	*R*_int_ = 0.070
*T* = 100.0(1) K	θ_max_ = 30.1º
φ and ω scans	θ_min_ = 2.8º
Absorption correction: multi-scan(SADABS; Bruker, 2005)	*h* = −12→9
*T*_min_ = 0.556, *T*_max_ = 0.715	*k* = −16→12
14370 measured reflections	*l* = −36→16

### Refinement

**Table:**

Refinement on *F*^2^	Secondary atom site location: difference Fourier map
Least-squares matrix: full	Hydrogen site location: inferred from neighbouring sites
*R*[*F*^2^ > 2σ(*F*^2^)] = 0.041	H-atom parameters constrained
*wR*(*F*^2^) = 0.090	*w* = 1/[σ^2^(*F*_o_^2^) + (0.0317*P*)^2^] where *P* = (*F*_o_^2^ + 2*F*_c_^2^)/3
*S* = 1.00	(Δ/σ)_max_ < 0.001
3893 reflections	Δρ_max_ = 0.43 e Å^−^^3^
164 parameters	Δρ_min_ = −0.54 e Å^−^^3^
Primary atom site location: structure-invariant direct methods	Extinction correction: none

### Special details

**Table:**

Experimental. The data was collected with the Oxford Cyrosystem Cobra low-temperature attachment.
Geometry. All e.s.d.'s (except the e.s.d. in the dihedral angle between two l.s. planes) are estimated using the full covariance matrix. The cell e.s.d.'s are taken into account individually in the estimation of e.s.d.'s in distances, angles and torsion angles; correlations between e.s.d.'s in cell parameters are only used when they are defined by crystal symmetry. An approximate (isotropic) treatment of cell e.s.d.'s is used for estimating e.s.d.'s involving l.s. planes.
Refinement. Refinement of *F*^2^ against ALL reflections. The weighted *R*-factor *wR* and goodness of fit *S* are based on *F*^2^, conventional *R*-factors *R* are based on *F*, with *F* set to zero for negative *F*^2^. The threshold expression of *F*^2^ > σ(*F*^2^) is used only for calculating *R*-factors(gt) *etc*. and is not relevant to the choice of reflections for refinement. *R*-factors based on *F*^2^ are statistically about twice as large as those based on *F*, and *R*- factors based on ALL data will be even larger.

### Fractional atomic coordinates and isotropic or equivalent isotropic displacement parameters (Å^2^)

**Table:**

	*x*	*y*	*z*	*U*_iso_*/*U*_eq_	
Br1	0.13862 (3)	0.42397 (2)	0.062535 (10)	0.02216 (9)	
O1	0.3082 (2)	0.38663 (17)	0.15935 (7)	0.0253 (5)	
C1	0.4236 (3)	0.6775 (2)	0.16809 (10)	0.0177 (6)	
H1A	0.3563	0.7084	0.1444	0.021*	
C2	0.4991 (4)	0.7501 (3)	0.20151 (10)	0.0228 (7)	
H2A	0.4813	0.8292	0.2005	0.027*	
C3	0.6008 (4)	0.7049 (3)	0.23638 (10)	0.0262 (7)	
H3A	0.6512	0.7537	0.2588	0.031*	
C4	0.6275 (4)	0.5867 (3)	0.23785 (10)	0.0266 (7)	
H4A	0.6977	0.5565	0.2608	0.032*	
C5	0.5499 (3)	0.5141 (3)	0.20533 (10)	0.0214 (6)	
H5A	0.5657	0.4348	0.2071	0.026*	
C6	0.4477 (3)	0.5591 (2)	0.16967 (9)	0.0158 (6)	
C7	0.3529 (3)	0.4759 (2)	0.13968 (9)	0.0159 (6)	
C8	0.3122 (3)	0.5048 (2)	0.08629 (9)	0.0144 (6)	
C9	0.3995 (3)	0.5748 (2)	0.05796 (9)	0.0143 (5)	
H9A	0.4848	0.6018	0.0754	0.017*	
C10	0.3931 (3)	0.6191 (2)	0.00603 (9)	0.0150 (6)	
C11	0.2836 (3)	0.5907 (2)	−0.03059 (10)	0.0188 (6)	
H11A	0.2098	0.5348	−0.0238	0.023*	
C12	0.2854 (3)	0.6461 (3)	−0.07730 (10)	0.0214 (6)	
H12A	0.2119	0.6268	−0.1013	0.026*	
C13	0.3942 (3)	0.7295 (2)	−0.08878 (9)	0.0198 (6)	
C14	0.5065 (4)	0.7535 (2)	−0.05306 (9)	0.0208 (6)	
H14A	0.5829	0.8070	−0.0605	0.025*	
C15	0.5059 (3)	0.6990 (2)	−0.00666 (9)	0.0176 (6)	
H15A	0.5825	0.7160	0.0166	0.021*	
C16	0.3901 (4)	0.7951 (3)	−0.13811 (10)	0.0304 (8)	
H16A	0.3307	0.7526	−0.1624	0.046*	
H16B	0.4928	0.8047	−0.1506	0.046*	
H16C	0.3444	0.8694	−0.1328	0.046*	

### Atomic displacement parameters (Å^2^)

**Table:**

	*U*^11^	*U*^22^	*U*^33^	*U*^12^	*U*^13^	*U*^23^
Br1	0.02062 (15)	0.02191 (16)	0.02395 (15)	−0.00743 (14)	−0.00516 (12)	0.00364 (12)
O1	0.0315 (13)	0.0193 (11)	0.0252 (11)	−0.0079 (10)	−0.0037 (9)	0.0057 (9)
C1	0.0193 (15)	0.0186 (15)	0.0152 (13)	0.0013 (13)	0.0009 (11)	0.0003 (11)
C2	0.0271 (17)	0.0233 (16)	0.0180 (13)	−0.0026 (14)	0.0031 (12)	−0.0054 (12)
C3	0.0308 (19)	0.0347 (19)	0.0130 (13)	−0.0064 (16)	−0.0013 (12)	−0.0074 (12)
C4	0.0237 (16)	0.039 (2)	0.0169 (13)	−0.0012 (17)	−0.0041 (12)	0.0032 (12)
C5	0.0280 (17)	0.0193 (16)	0.0170 (13)	0.0043 (14)	0.0008 (13)	0.0025 (11)
C6	0.0167 (14)	0.0197 (16)	0.0110 (12)	0.0007 (12)	0.0033 (10)	0.0019 (10)
C7	0.0152 (14)	0.0149 (14)	0.0177 (13)	0.0020 (12)	0.0008 (11)	0.0013 (11)
C8	0.0141 (13)	0.0136 (14)	0.0155 (13)	−0.0018 (11)	−0.0013 (11)	−0.0022 (10)
C9	0.0121 (13)	0.0138 (13)	0.0168 (12)	0.0022 (12)	−0.0004 (10)	−0.0039 (11)
C10	0.0198 (16)	0.0109 (13)	0.0142 (12)	0.0038 (12)	−0.0001 (11)	−0.0029 (10)
C11	0.0184 (15)	0.0199 (16)	0.0181 (13)	0.0010 (13)	0.0005 (11)	−0.0011 (11)
C12	0.0199 (16)	0.0273 (17)	0.0171 (13)	0.0038 (14)	−0.0025 (11)	−0.0013 (12)
C13	0.0250 (17)	0.0218 (15)	0.0125 (13)	0.0074 (13)	0.0041 (11)	0.0027 (11)
C14	0.0274 (17)	0.0174 (15)	0.0177 (13)	−0.0021 (13)	0.0047 (12)	0.0022 (11)
C15	0.0214 (15)	0.0157 (15)	0.0157 (13)	−0.0024 (13)	−0.0004 (11)	−0.0033 (11)
C16	0.036 (2)	0.0352 (19)	0.0198 (14)	0.0072 (16)	−0.0008 (13)	0.0096 (13)

### Geometric parameters (Å, °)

**Table:**

Br1—C8	1.888 (3)	C9—C10	1.469 (3)
O1—C7	1.221 (3)	C9—H9A	0.9300
C1—C2	1.386 (4)	C10—C15	1.391 (4)
C1—C6	1.388 (4)	C10—C11	1.400 (4)
C1—H1A	0.9300	C11—C12	1.394 (4)
C2—C3	1.383 (4)	C11—H11A	0.9300
C2—H2A	0.9300	C12—C13	1.387 (4)
C3—C4	1.390 (4)	C12—H12A	0.9300
C3—H3A	0.9300	C13—C14	1.389 (4)
C4—C5	1.381 (4)	C13—C16	1.511 (4)
C4—H4A	0.9300	C14—C15	1.382 (3)
C5—C6	1.399 (4)	C14—H14A	0.9300
C5—H5A	0.9300	C15—H15A	0.9300
C6—C7	1.497 (4)	C16—H16A	0.9600
C7—C8	1.496 (3)	C16—H16B	0.9600
C8—C9	1.341 (4)	C16—H16C	0.9600
			
C2—C1—C6	120.5 (3)	C10—C9—H9A	112.3
C2—C1—H1A	119.7	C15—C10—C11	118.1 (2)
C6—C1—H1A	119.7	C15—C10—C9	115.5 (2)
C3—C2—C1	120.1 (3)	C11—C10—C9	126.3 (3)
C3—C2—H2A	120.0	C12—C11—C10	119.9 (3)
C1—C2—H2A	120.0	C12—C11—H11A	120.1
C2—C3—C4	119.9 (3)	C10—C11—H11A	120.1
C2—C3—H3A	120.0	C13—C12—C11	121.5 (3)
C4—C3—H3A	120.0	C13—C12—H12A	119.2
C5—C4—C3	120.0 (3)	C11—C12—H12A	119.2
C5—C4—H4A	120.0	C12—C13—C14	118.2 (2)
C3—C4—H4A	120.0	C12—C13—C16	121.5 (3)
C4—C5—C6	120.4 (3)	C14—C13—C16	120.3 (3)
C4—C5—H5A	119.8	C15—C14—C13	120.7 (3)
C6—C5—H5A	119.8	C15—C14—H14A	119.6
C1—C6—C5	119.0 (3)	C13—C14—H14A	119.6
C1—C6—C7	122.4 (2)	C14—C15—C10	121.4 (3)
C5—C6—C7	118.0 (2)	C14—C15—H15A	119.3
O1—C7—C8	121.1 (2)	C10—C15—H15A	119.3
O1—C7—C6	119.7 (2)	C13—C16—H16A	109.5
C8—C7—C6	119.2 (2)	C13—C16—H16B	109.5
C9—C8—C7	122.0 (2)	H16A—C16—H16B	109.5
C9—C8—Br1	124.7 (2)	C13—C16—H16C	109.5
C7—C8—Br1	113.19 (19)	H16A—C16—H16C	109.5
C8—C9—C10	135.5 (3)	H16B—C16—H16C	109.5
C8—C9—H9A	112.3		
			
C6—C1—C2—C3	0.8 (4)	C6—C7—C8—Br1	−158.5 (2)
C1—C2—C3—C4	0.1 (4)	C7—C8—C9—C10	179.8 (3)
C2—C3—C4—C5	−1.5 (4)	Br1—C8—C9—C10	5.1 (5)
C3—C4—C5—C6	2.0 (4)	C8—C9—C10—C15	175.3 (3)
C2—C1—C6—C5	−0.3 (4)	C8—C9—C10—C11	−3.2 (5)
C2—C1—C6—C7	171.0 (3)	C15—C10—C11—C12	−3.1 (4)
C4—C5—C6—C1	−1.1 (4)	C9—C10—C11—C12	175.3 (3)
C4—C5—C6—C7	−172.8 (3)	C10—C11—C12—C13	0.3 (4)
C1—C6—C7—O1	−136.9 (3)	C11—C12—C13—C14	2.5 (4)
C5—C6—C7—O1	34.5 (4)	C11—C12—C13—C16	−176.0 (3)
C1—C6—C7—C8	42.0 (4)	C12—C13—C14—C15	−2.4 (4)
C5—C6—C7—C8	−146.6 (3)	C16—C13—C14—C15	176.1 (3)
O1—C7—C8—C9	−154.8 (3)	C13—C14—C15—C10	−0.5 (4)
C6—C7—C8—C9	26.3 (4)	C11—C10—C15—C14	3.2 (4)
O1—C7—C8—Br1	20.4 (3)	C9—C10—C15—C14	−175.3 (2)

### Hydrogen-bond geometry (Å, °)

**Table:**

*D*—H···*A*	*D*—H	H···*A*	*D*···*A*	*D*—H···*A*
C1—H1A···O1^i^	0.93	2.54	3.163 (3)	124
C11—H11A···Br1	0.93	2.69	3.377 (3)	131

Symmetry codes: (i) −*x*+1/2, *y*+1/2, *z*.
