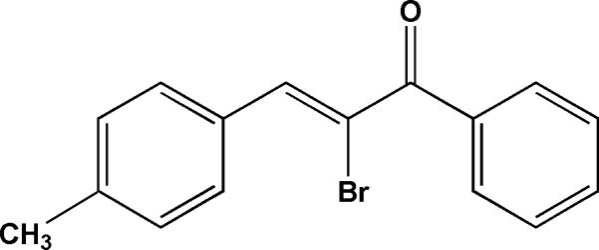# 2-Bromo-1-(4-methyl­phen­yl)-3-phenyl­prop-2-en-1-one. Corrigendum

**DOI:** 10.1107/S1600536808035356

**Published:** 2008-11-13

**Authors:** Hoong-Kun Fun, Samuel Robinson Jebas, P. S. Patil, M. S. Karthikeyan, S. M. Dharmaprakash

**Affiliations:** a X-ray Crystallography Unit, School of Physics, Universiti Sains Malaysia, 11800 USM, Penang, Malaysia; b Department of Studies in Physics, Mangalore University, Mangalagangotri, Mangalore 574 199, India; c Syngene International Pvt Limited, Plot Nos. 2 and 3 C, Unit-II, Bommansandra, Industrial Area, Banglore 560 099, India

## Abstract

The chemical name in the title and the scheme of the paper by Fun, Jebas, Patil, Karthikeyan & Dharmaprakash [*Acta Cryst.* (2008), E**64**, o1559] are corrected.

## Abstract 2

Corrigendum to *Acta Cryst.* (2008), E**64**, o1559.

In the paper by Fun, Jebas, Patil, Karthikeyan & Dharmaprakash [*Acta Cryst.* (2008), E**64**, o1559], the chemical name in the title and the scheme are incorrect. The correct title should be ‘2-Bromo-3-(4-methyl­phen­yl)-1-phenyl­prop-2-en-1-one’ and the correct scheme is shown below.